# Does Cryopreservation of Ovarian Tissue Affect the Distribution and Function of Germinal Vesicle Oocytes Mitochondria?

**DOI:** 10.1155/2013/489032

**Published:** 2013-07-17

**Authors:** Mojdeh Salehnia, Virpi Töhönen, Saeed Zavareh, Jose Inzunza

**Affiliations:** ^1^Anatomy Department, Tarbiat Modares University, P.O. Box 14115-111, Tehran, Iran; ^2^Division of Medical Nutrition, Department of Biosciences and Nutrition, Karolinska University Hospital, Karolinska Institutet, Novum, 141 86 Stockholm, Sweden; ^3^School of Biology, Damghan University, P.O. Box 3671641167, Damghan, Iran

## Abstract

The aim of this study was to evaluate mitochondrial alteration and ATP content of germinal vesicle (GV) oocytes isolated from fresh and vitrified ovaries. After superovulation, the ovaries from adult mice were collected and divided into control and vitrified groups. GV oocytes were isolated mechanically from each group. Half were cultured for 24 hours and their maturation was assessed. Metaphase II oocytes were collected and submitted to *in vitro* fertilization and their fertilization rates and development to the blastocyst stage were evaluated. In the remaining GV oocytes, ATP levels were quantified, and mitochondrial distribution, mitochondrial membrane potential, and intracellular free calcium were detected with rhodamine 123, JC-1 and Flou-4 AM staining, using laser-scanning confocal microscopy. Maturation and fertilization rates of GV oocytes and the developmental rates of subsequent embryos were significantly lower in vitrified samples (*P* < 0.05). The ATP content and Ca^2+^ levels differed significantly in fresh and vitrified GV oocytes (*P* < 0.05). Most mitochondria were seen as large and homogenous aggregates (66.6%) in fresh GV oocytes compared to vitrified oocytes (50%). No significant differences in mitochondrial membrane potential were found between the groups. The lower maturation and fertilization rates of GV oocytes from vitrified ovaries may be due to changes in their mitochondrial function and distribution.

## 1. Introduction

Vitrification is a method for cryopreservation of oocytes, embryos, and ovarian tissue. The survival and development rates of vitrified oocytes, embryo, and follicles have been studied [1–4]. The vitrified oocytes and follicles have been found to mature somewhat more slowly than fresh samples [2–6]. However, the further development of vitrified follicles compared with nonvitrified samples is controversial [2, 3, 6–8].

Cryopreservation causes alterations in the physical and chemical properties of oocytes and embryos, including loss of cell membrane and cytoskeletal integrity, mitochondrial depolarization, and increased production of reactive oxygen species [9–15]. These alterations are associated with osmotic forces created during dehydration, cooling, rehydration, and warming and may affect mainly cytoplasmic activities such as mitochondrial function, metabolism, and intracellular signaling pathways.

The number and distribution of mitochondria and energy (ATP) production are critical factors that influence not only the maturation and development of the oocyte but also its fertilization and subsequent embryo development [16–20]. Structural and metabolic mitochondrial defects are associated with failures in oocyte maturation and abnormal development or arrest of embryos [21–24]. Reports on vitrification methods using different cryoprotectants show that mitochondrial distribution is affected in mouse metaphase II (MII) oocytes and embryo at two pronucleus stages [11, 12, 15]. However, impairment of mitochondrial function by vitrification was shown in bovine and human oocytes [14, 25]. Recently, Demant et al. showed transient changes in mouse oocyte mitochondrial activity after vitrification [11].

*In vitro* maturation of isolated follicles and germinal vesicle (GV) oocytes from vitrified ovarian tissue may be an alternative technique for preserving fertility potential in parallel with oocyte preservation. Several previous reports were focused on vitrification of single oocytes or follicles, then their development was assessed [1–4] whereas, to our knowledge, no research has documented the effects of ovarian vitrification on the maturation and distribution of mitochondria and ATP content of isolated GV oocytes.

Dysfunction of oocyte mitochondria may occur without detectable morphological abnormalities. Therefore, we studied the effects of ovarian vitrification on mitochondrial distribution and function in mouse GV oocytes using mitochondria-specific fluorescent probes. The energy (ATP) and free Ca^2+^ concentrations and the developmental potential of the GV oocytes were also evaluated.

## 2. Materials and Methods 

All chemicals were purchased from Sigma-Aldrich (St. Louis, USA), unless otherwise noted.

### 2.1. Animals and Collection of Ovarian Tissue

Adult female FVB/N mice, 6–8 weeks' old, were housed under controlled conditions (12 h light/12 h dark) with free access to water and food. The mice were superovulated using an i.p. injection of 10 IU pregnant mare serum gonadotropin. After 48 h, superovulated female mice were sacrificed by cervical dislocation and ovaries were dissected, freed of fat and mesentery, and divided into control (nonvitrified) and vitrified groups. Animal experiments were performed at the infection-free animal facility of Karolinska University Hospital in accordance with the ethical committee's approval.

### 2.2. Vitrification and Warming

The vitrification procedure was based on a method used earlier [26]. Briefly, ovarian tissue was equilibrated in a vitrification medium (EFS40) containing 40% ethylene glycol (V/V), 30% Ficoll 70 (W/V), and 1 M sucrose supplemented with bovine serum albumin (BSA) for 5 minutes at room temperature. The ovaries were placed in plastic cryotubes with a minimum volume of vitrification medium (10 *µ*L), then put on nitrogen vapor for 20 seconds, immersed in liquid nitrogen, and maintained for 1 week. Vitrified ovaries were warmed at room temperature and placed in a 25°C water bath for 20 seconds. The contents of each cryotube were placed in 1 mL of descending concentrations of sucrose (1 and 0.5 M) at room temperature for 5 minutes. Warmed ovaries were equilibrated for 30 minutes in tissue culture medium (TCM 199; Gibco, Grand Island, NY, USA) supplemented with 5 mg/mL BSA before isolation and collection of GV oocytes.

### 2.3. Isolation and Collection of the Germinal Vesicle Oocytes

The GV oocytes (*n* = 441 for fresh and 328 for vitrified groups) were mechanically isolated from large preantral follicles from fresh and vitrified-thawed ovaries using 29 gauge needles during five minutes. Oocytes with a prominent GV and clear ooplasm (average 90 *µ* diameter) were selected for observation using a stereomicroscope. The survival rate of isolated oocytes was assessed morphologically. Collected oocytes were used for the following experiments.

### 2.4. *In Vitro* Maturation of GV Oocytes

The maturation medium TCM199 was supplemented with 50 *μ*g/mL penicillin, 75 *μ*g/mL streptomycin, 0.23 mM sodium pyruvate, 10% fetal bovine serum, 75 mIU/mL rFSH, and 10 IU/mL hCG (Serono, Switzerland). GV oocytes (*n* = 261 for control and 183 for vitrified group) were cultured in 30 *µ*L drops of maturation medium under mineral oil at 37°C, 100% humidity in 5% CO_2_ for 18 h. At the end of the culture period, the number of oocytes at the GV stage, germinal vesicle breakdown (GVBD), and MII was counted using an inverted microscope. MII oocytes were collected and used for *in vitro* fertilization.

### 2.5. *In Vitro* Fertilization and Embryo Culture

Cauda epididymes were dissected from mature male mice and placed into 500 *µ*L of T6 medium with 5 mg/mL BSA under mineral oil drops containing freshly released spermatozoa were placed in 37°C and 5% CO_2_ incubator for capacitation.

The collected MII oocytes from control (*n* = 226) and vitrified (*n* = 92) groups were transferred to T6 medium supplemented with 15 mg/mL BSA and containing capacitated spermatozoa. 4–6 hours later, the oocytes were transferred to 10 *µ*L drops of T6 medium with 5 mg/mL BSA. The presence of 2 pronucleus zygote was considered as successful fertilization. Embryos were observed daily under an inverted microscope, and the number of embryos reaching 2-cell, 4-cell, morula, and blastocyst stages was recorded for 120 hours [27].

### 2.6. Measurement of Cytoplasmic ATP Content

The ATP content of oocytes was measured using methods described previously [28]. Briefly, GV stage oocytes were isolated from control (*n* = 30) and vitrified samples (*n* = 25) and rapidly frozen individually in microtubes containing 200 *µ*L of ultrapure water at −80°C. ATP level of each single oocyte was quantified by measuring luminescence (Berthold LB 9501 luminometer) in an ATP-dependent luciferin-luciferase bioluminescence assay (Bioluminescence Somatic Cell Assay System; Sigma, USA).

A standard curve with different ATP concentrations was generated for each series of analyses. ATP content was determined by the standard curve.

### 2.7. Visualization of Mitochondria Using Rhodamine 123 (R123)

Viable mitochondria were identified using R123 (Molecular Probes, Invitrogen, Eugene, OR, USA) staining as described by Van Blerkom et al. [29]. A stock solution of 10 *µ*g/mL R123 in dimethylsulfoxide was prepared and stored at −20°C. GV oocytes from both study groups (at least 20 oocytes per each group) were stained with 1 *µ*g/mL R123 in TCM medium for 10 min in the dark at 37°C. After washing samples in TCM medium, oocytes were visualized using LSCM (Olympus, Segrate, Italy) to detect and capture serial sections of oocytes for assessment of cytoplasmic distribution of R123 (520 nm emission).

### 2.8. Intracellular Free Calcium Analysis by Fluo-4 AM

The free Ca^2+^ content of the oocytes was detected by LSCM as average relative fluorescence intensity (RFI) in individual fresh (*n* = 20) and vitrified GV oocytes (*n* = 25) according to Jones et al.'s [9] protocol. Briefly, oocytes were preloaded for 60 min in TCM medium supplemented with 4% BSA and 20 *µ*mol/L Fluo-4 AM (Molecular Probes, Invitrogen, Eugene, OR, USA), followed by a 30 min wash in normal medium. Oocytes were transferred to an LSCM chamber maintained at 37°C. For quantifying relative RFI, a long path filter with an emission detection >510 nm was used and digital images were processed.

### 2.9. Determination of Mitochondrial Membrane Potential (∆Ψ) by JC-1

Mitochondrial potential assays were performed according to the method of Smiley et al. [30]. The potential sensitive fluorescence dye JC-1 (5,5′6,6′-tetrachloro-1,1,3,3′-tetraethylbenzimidazolycarbocyanine iodide (Molecular Probes, Invitrogen, Eugene, OR, USA) was used to measure the activity of oocyte mitochondria.

JC-1 dye was dissolved in dimethylsulfoxide at 10 *µ*g/mL as a stock solution and diluted into preequilibrated TCM medium (Sigma) at 1 *µ*g/mL, using a vortex. GV oocytes were isolated from fresh (*n* = 20 GV) and vitrified (*n* = 15 GV) ovaries, exposed to the dye at 37°C in a 5% CO_2_ incubator for 10 min in TCM medium, and then washed in TCM medium. The relative fluorescence intensity of JC-1 stained oocytes was determined by LSCM. The distribution of JC-1 monomer (green fluorescence detected in the fluorescein isothiocyanate channel, 515–530 nm bandpass filter) and J-aggregate fluorescence (orange/red J-aggregate fluorescence) was detected in the rhodamine isothiocyanate channel, using 585 nm long path filter. Laser power and photomultiplier settings were kept constant for all experiments. Images of individual zygotes were captured and quantified for fluorescence intensity.

### 2.10. Detection of ROS Content in GV Oocytes Using Spectrofluorometer

To measure ROS levels in GV oocytes derived from vitrified (*n* = 100) and nonvitrified (*n* = 100) mouse ovaries, after mechanical isolation of oocytes from ovaries, 20 oocytes were pooled in each experiment (*n* = 60 in five repeats in each group), washed 3 times with phosphate buffer saline (PBS), and incubated in 40 mmol/L of Tris-HCl buffer (pH = 7.0) containing 5 *μ*mol/L 2′, 7′-dichlorodihydrofluorescein diacetate (DCHFDA; Merck; Darmstadt, Germany) at 37°C for 30 min. Then, the solution was removed and the oocytes were washed three times with PBS, immediately homogenized in 100 *μ*L of Tris-HCl buffer (40 mmol/L, pH 7.0), sonicated at 50 W for 1 min, and centrifuged at 10,000 ×g for 20 min at 4°C, and the supernatants were collected. Fluorescence was monitored in the supernatant using a spectrofluorometer at 488 nm excitation and at 525 nm emission [31]. Data were expressed as *μ*M H_2_O_2_ and the mean dichlorofluorescein (DCF) fluorescence intensity (means ± SEM). The analysis for each sample was done duplicately.

### 2.11. Statistical Analysis

All experiments were repeated at least three times. Oocyte maturation and embryo development were analyzed by one-way ANOVA, and Tukey's HSD was used as post hoc tests. ATP levels and RFI values for JC-1 and Fluo-4 AM were analyzed statistically by the *t-*test and were considered significant at *P* < 0.05.

## 3. Results

### 3.1. Maturation Rates of Vitrified and Nonvitrified Tissue-Isolated GV Oocytes

The rates of morphologically normal isolated GV oocytes derived from fresh and vitrified mouse ovaries were 97% and 89%, respectively. No significant differences were observed in the proportion of normal oocytes between the two groups.

Table 1 summarizes the maturation and fertilization rates of the GV oocytes and their subsequent embryo development. The MII stage was reached by 86.9% ± 4.51 of GV oocytes in the control group and 50.20% ± 6.97 in the vitrified group. The rates in the vitrified samples were lower than those in controls (*P* < 0.05).

Fertilization rates were 80.97% ± 3.46 in the control group and 57.30% ± 10.85 in the vitrified group, and blastocyst formation rates were 40.98 ± 2.74 and 11.36% ± 5.51 in control and vitrified groups, respectively. The rates in the vitrified samples were lower than those in controls (*P* < 0.05).

### 3.2. ATP Content of Tissue-Isolated GV Oocytes

**Figure 1:**
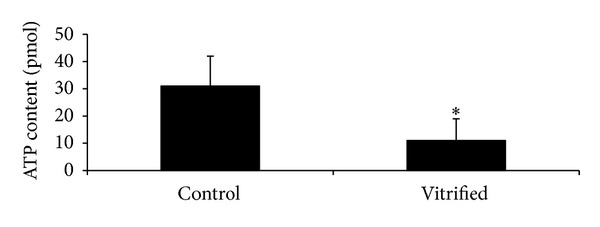
ATP content (pmol) of GV oocytes from fresh and vitrified mouse ovaries. *Significant difference between groups (*P* < 0.05).

As shown in Figure 1, the ATP content of individual GV oocytes derived from vitrified samples (11 × 10^−12^ ± 8 × 10^−12^ mol) was significantly lower (*P* < 0.05) than that of fresh samples (31 × 10^−12^ ± 11 × 10^−12^ mol).

### 3.3. Mitochondrial Distribution

**Figure 2:**
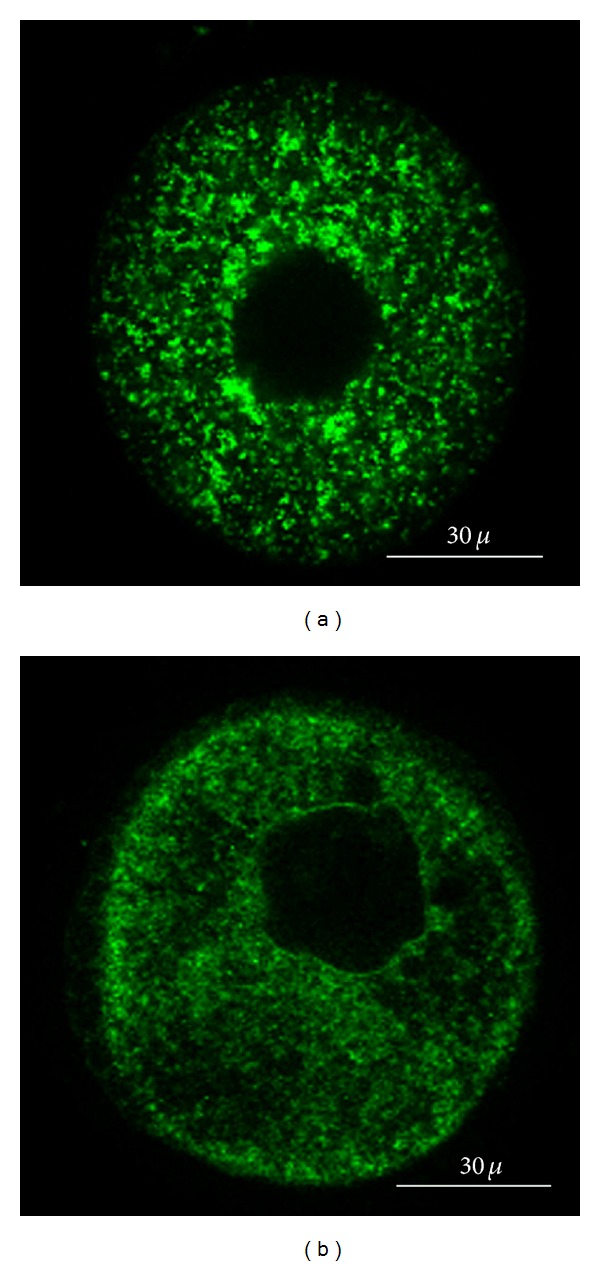
Distribution of viable mitochondria stained by R123. The mitochondrial pattern was (a) uniform and large aggregates or (b) small aggregates.

Laser scanning confocal microscopy (LSCM) analysis of GV oocytes loaded with rhodamine 123 (R123) showed two distinct mitochondrial distribution patterns in the ooplasm (Figures 2(a) and 2(b)): uniform, large aggregates and uniform, small aggregates. 66.6% of the GV oocytes from fresh tissue had large aggregates, and 33.3% had small aggregates. In the vitrified group, 50% of the oocytes showed large aggregates, and 50% showed small ones.

### 3.4. Intracellular Free Calcium in Tissue-Isolated GV Oocytes

**Figure 3:**
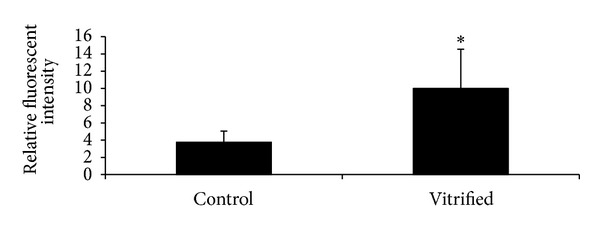
Cytoplasmic free calcium levels measured by relative fluorescence intensity with Flou-4 AM staining in control and vitrified groups of GV oocytes. *Significant difference between groups (*P* < 0.05).

Cytoplasmic free calcium concentrations were quantified using Flou-4 AM staining. The relative fluorescence intensity was 3.753 ± 1.29 in fresh samples and 10 ± 4.55 in vitrified samples (Figure 3). Differences between the vitrified and fresh groups were significant (*P* < 0.05).

### 3.5. Mitochondrial Membrane Potential (∆Ψ) of Tissue-Isolated GV Oocytes

**Figure 4:**
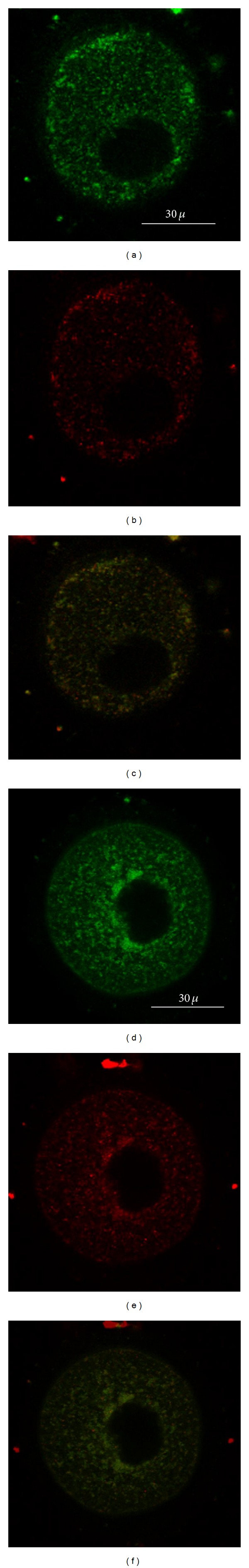
The confocal images of GV oocytes were stained with JC-1. Green and red mitochondria were viewed at 515–530 and 585 nm, respectively. Fresh ((a)–(c)) and vitrified GV oocytes ((d)–(f)): the figures were merged at third column ((c) and (f)). Green (JC-1 monomers) indicating low-polarized mitochondria and red (J-aggregates) denoting high-polarized mitochondria.

**Figure 5:**
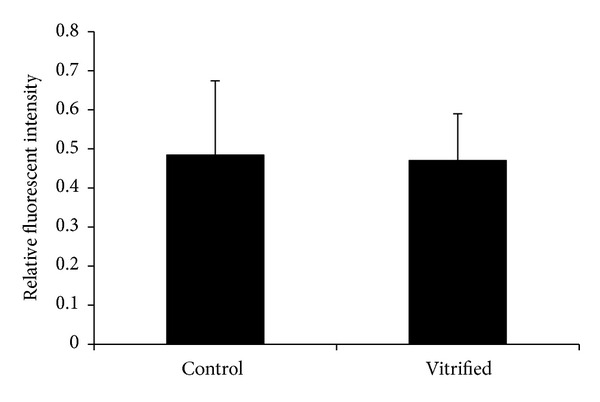
Mitochondrial membrane potential calculated using the ratio of relative fluorescence intensity of red to green in JC-1 stained GV oocytes from fresh and vitrified ovaries.

To detect mitochondrial membrane potential, GV oocytes were stained with JC-1 monomer. Oocytes exhibited slightly punctate J-aggregate fluorescence (Figure 4). The red/green ratio of relative fluorescence intensity was 0.484 ± 0.19 in fresh samples and 0.47 ± 0.12 in vitrified samples (Figure 5), which was not a significant difference.

### 3.6. ROS Content in GV Oocytes

**Figure 6:**
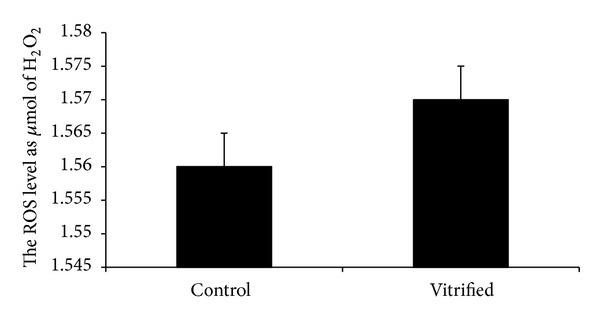
Reactive oxygen species (ROS) levels in GV oocytes derived from nonvitrified (control) and vitrified mouse ovaries. There was no significant difference (data as mean ± SD).

ROS levels in GV oocytes derived from non-vitrified and vitrified ovarian tissue are shown in Figure 6. The level of ROS in vitrified (1.57 ± 0.017 *µ*mol) and non-vitrified (1.56 ± 0.020 *µ*mol) samples was not statistically different.

## 4. Discussion

Vitrification of ovarian tissue is an alternative method for preserving the fertility of cancer patients before they undergo chemotherapy or radiotherapy [32]. Cryopreservation of immature (GV) and MII oocytes, in combination with ovarian tissue vitrification, may be effective for fertility preservation. Vitrification of oocytes at different developmental stages has been successful [33], but direct vitrification of follicles was showed to be better than the vitrification of isolated follicles derived from ovarian tissue [3]. However, data on the developmental rates of immature (GV) oocytes collected from vitrified ovarian tissue is limited, thus, this study has focused on this subject.

We show here that vitrified mouse ovaries yielded a high proportion of morphologically normal GV oocytes; however, their *in vitro* development and fertilization rates were lower than oocytes from nonvitrified ovaries. Our previous reports and other studies show that the morphology and ultrastructure of mouse oocytes and follicular cells are well preserved after vitrification and subsequent warming of ovarian tissue [7, 26, 27, 34]. However, the low fertilization and developmental rates of vitrified GV oocytes could be due to changes that are not visible at light and electron microscopic levels or some changes which need more time to affect the development of oocyte. On the other hand, the vitrified oocytes may be more sensitive than non-vitrified samples to mechanical isolation procedure and/or the damages which were created during the isolation technique which affect their development.

In this study, we focused on oocyte mitochondrial changes after vitrification and warming. Mitochondria are the critical organelles in the oocyte and contribute to several important cell functions including ATP production. We analyzed mitochondria biochemically to understand some of these changes during the cooling and warming procedure.

Our results demonstrated that ATP concentration was significantly reduced in vitrified samples compared with oocytes from fresh tissue. Inadequate ATP production in vitrified GV oocytes may result in delayed and decreased development and maturation of oocytes. Similar results were reported by Gualtieri et al. [35] using slow cooling cryopreservation of human MII oocytes. They demonstrated mitochondrial damage and reduced developmental competence of cryopreserved oocytes.

Specific events during embryo development are ATP dependent including pronucleus formation, syngamy, embryo genome activation, successive cell cleavage, compaction, lineage differentiation, and blastocoel formation [36, 37]. Therefore, a reduction in the ATP content of vitrified GV oocyte could be a reason for impairment of oocyte fertilization and embryo development.

Furthermore, the localization of mitochondria within the ooplasm is important for oocyte development. Mitochondria are mainly localized in regions that have more energy requirements [36, 38–40]. Our LSCM observation showed that the mitochondrial distribution pattern differed between fresh and vitrified groups. The reduction in ATP content of vitrified samples might influence microtubule-mediated mitochondrial reorganization. It was suggested that altered mitochondrial distribution affected the developmental potential of vitrified mouse ovary-derived oocytes [41].

An earlier study demonstrated that mouse GV oocytes with abnormal mitochondrial distribution fail to develop to the MII stage [41] and small foci of mitochondria at the cortical part of GV oocytes are characteristic of arrested oocytes [39, 42].

Nagai et al. [41] demonstrated mitochondrial dysfunction and fragmentation in mouse MII oocytes after vitrification and warming. They concluded that an abnormal mitochondrial distribution in MII oocytes is one of the causes of developmental retardation and arrest. Vitrification has been suggested to alter important parameters of mitochondria distribution and organization in porcine MII oocytes [43].

Mitochondrial membrane potential is an indication of mitochondrial function [44]. Our LSCM observation using JC-1 staining showed that the ∆Ψ was similar in both study groups. Hence, ovarian tissue vitrification did not affect the mitochondrial polarity of mouse GV oocytes, but recently Demant et al. demonstrated that mitochondrial inner membrane potential differed significantly between control and vitrified GV oocytes; however, it was similar after 12 days of culture [11].

As we demonstrated that in other part of this study the ATP levels in vitrified samples were lower than the fresh groups, the mitochondrial inner membrane potential was not different in these groups. One explanation for this observation may be due to a reduction in the ATP synthesis within the ooplasm; however, it needs more study.

Mitochondria are important regulators of free intracellular calcium homeostasis. In this study, we showed for the first time that the intracellular free calcium in vitrified GV oocytes increased compared to non-vitrified samples. This could be due to damage to the cell membrane or mitochondria during vitrification. An increase in intracellular calcium during vitrification could be due to influx from outside the cell after damage to the cell membrane or could come from mitochondria or the endoplasmic reticulum, which are intracellular calcium stores [45–48]. Lowther et al. reported that disruption of endoplasmic reticulum during vitrification of mouse oocytes and deficiencies in factors involved in endoplasmic reticulum reorganization during oocyte maturation could contribute to the low development potential of vitrified *in vitro*-matured oocytes [49].

Successful fertilization and embryo cleavage are both ATP and calcium dependent. Changes in their levels could affect not only fertilization rate, but also embryonic development [49–51]. An increase in oocyte calcium during vitrification could be involved in premature exocytosis of cortical granules resulting in zona pellucida hardening that prevents sperm penetration. A similar observation was reported after exposure of oocytes to cryoprotectant solution [52, 53].

However, in other part of this study, our result showed that there was no significant increase in the ROS level of vitrified samples in comparison with their control. It means that, in spitve of some changes in the mitochondria of vitrified GV, these alterations could not cause an increase in oxidative stress. Furthermore, the high level of ROS could induce apoptotic cell death by mitochondrial apoptotic pathways [54].

However, this study was focused on the GV oocytes and more studies need to compare the effects of this cryopreservation technique on the MII stage oocyte.

## 5. Conclusion

In conclusion, vitrification of mouse ovaries using the vitrification medium EFS40 changed the mitochondrial distribution, reduced the ATP content, and increased intracellular free calcium in isolated ovarian GV oocytes. However, vitrification had no effect on mitochondrial membrane polarity. These changes could cause delays in maturation and impede the development and survival of GV oocytes.

## Figures and Tables

**Table 1:** The maturation rates of the mouse vitrified and nonvitrified tissue-isolated GV oocytes.

Groups	No. of isolated GV	No. of GV reached MII	No. of degenerated	No. of fertilized	No. of 2 cell	No. of 8 cell	No. of morula	No. of blastocyst
Control	261	226 (86.9 ± 4.51)	6 (2.19 ± 1.01)	183 (81 ± 3.46)	163 (89 ± 4.24)	100 (54.6 ± 3.79)	86 (46.9 ± 3.82)	75 (40.9 ± 2.74)
Vitrification	183	92* (50.2 ± 6.97)	30* (16.3 ± 6.5)	44* (47.8 ± 10.85)	28* (63.6 ± 8.42)	12* (27.2 ± 4.88)	7* (15.9 ± 7.64)	5* (11.36 ± 5.51)

Four experimental replicates were performed for each group. GV: germinal vesicle stage oocyte; MII: metaphase II oocyte. Data within prentices are % ± SD. *Significant differences compared to the control group in the same column (*P* < 0.05).
